# SEPTIN2 and STATHMIN Regulate CD99-Mediated Cellular Differentiation in Hodgkin's Lymphoma

**DOI:** 10.1371/journal.pone.0127568

**Published:** 2015-05-22

**Authors:** Wenjing Jian, Lin Zhong, Jing Wen, Yao Tang, Bo Qiu, Ziqing Wu, Jinhai Yan, Xinhua Zhou, Tong Zhao

**Affiliations:** 1 Department of Molecular and Tumor Pathology Laboratory of Guangdong Province, School of Basic Medical Science, Southern Medical University, Guangzhou, China; 2 Department of Pathology, the Third Affiliated Hospital, Southern Medical University, Guangzhou, China; The Walter and Eliza Hall of Medical Research, AUSTRALIA

## Abstract

Hodgkin’s lymphoma (HL) is a lymphoid neoplasm characterized by Hodgkin’s and Reed-Sternberg (H/RS) cells, which is regulated by *CD99*. We previously reported that *CD99* downregulation led to the transformation of murine B lymphoma cells (A20) into cells with an H/RS phenotype, while *CD99 *upregulation induced differentiation of classical Hodgkin’s lymphoma (cHL) cells (L428) into terminal B-cells. However, the molecular mechanism remains unclear. In this study, using fluorescence two-dimensional differential in-gel electrophoresis and matrix-assisted laser desorption/ionization time of flight mass spectrometry (MALDI-TOF MS), we have analyzed the alteration of protein expression following *CD99* upregulation in L428 cells as well as downregulation of mouse *CD99* antigen-like 2 (m*CD99*L2) in A20 cells. Bioinformatics analysis showed that *SEPTIN2* and *STATHMIN*, which are cytoskeleton proteins, were significantly differentially expressed, and chosen for further validation and functional analysis. Differential expression of *SEPTIN2* was found in both models and was inversely correlated with *CD99* expression. *STATHMIN* was identified in the A20 cell line model and its expression was positively correlated with that of *CD99*. Importantly, silencing of *SEPTIN2* with siRNA substantially altered the cellular cytoskeleton in L428 cells. The downregulation of *STATHMIN* by siRNA promoted the differentiation of H/RS cells toward terminal B-cells. These results suggest that *SEPTIN2*-mediated cytoskeletal rearrangement and *STATHMIN*-mediated differentiation may contribute to changes in cell morphology and differentiation of H/RS cells with *CD99* upregulation in HL.

## Introduction

Hodgkin’s lymphoma (HL) is one of the most common malignant neoplasms affecting the lymphoid and hematological systems. Classical Hodgkin’s lymphoma (cHL) is characterized by Hodgkin cells and multinucleated Reed-Sternberg cells (H/RS) [1]. Accumulating evidence suggests that H/RS cells are derived from clonal B-cells with loss of their B-cell phenotype [2]. Mature B-cells lacking B-cell receptors (BCR) normally die via apoptosis, suggesting that H/RS cells must have developed mechanisms to maintain survival. H/RS cells present a complex immunophenotype. For example, H/RS cells usually express markers associated with the myeloid lineage (CD15) and markers associated with plasma cells (CD138, MUM-1) [3, 4], but rarely B-cell markers, such as CD20, Oct-2, Ig, or components of the BCR (*CD79a* and *CD79b*) [5]. The cause of the aberrant expression of a large number of B-cell genes is currently unclear. It is proposed that B-cell markers are lost due to aberrant gene regulation and cytoskeletal rearrangements [6]. H/RS cells are also characterized by abortive plasma cell differentiation [7], although, the underlying molecular mechanism is largely unknown.

*CD99*, a transmembrane glycoprotein encoded by the *MIC2* gene, is broadly expressed in hematopoietic cells, such as B-cells, T-cells, mononuclear cells, and neutrophils [8]. *CD99* is highly expressed in non-Hodgkin lymphoma, including acute lymphoblastic lymphoma [9], but rarely expressed in H/RS cells in cHL, with the mechanism still elusive. Several studies indicate that the generation of H/RS-like cells might be related to the downregulation of *CD99* [10, 11]. Kim et al [12] transfected IM9 (Ig-secreting lymphoblast) and BJAB (Burkitt’s lymphoma) cell lines with antisense *CD99* and found that downregulation of *CD99* led to the generation of cells with an H/RS phenotype. We previously reported that upregulation of *CD99* in L428 cell line (L428-*CD99*) resulted in loss of H/RS cells morphology [13]. In addition, downregulation of mouse *CD99* antigen-like 2 (m*CD99*L2) in murine A20 cells led to induction of some H/RS-cell like morphologies [14]. The m*CD99*L2 antigen shows sequence homology to human *CD99* [15]. A20 is a murine cell line derived from a spontaneously arising tumor in an aged BALB/c mouse with the characteristic pathology of human diffuse large B-cell lymphoma (DLBCL) [16, 17]. Taken together, these findings suggest that *CD99* plays a critical role in H/RS cellular differentiation.

To investigate the underlying mechanism by which *CD99* regulates H/RS cell differentiation, we used two-dimensional differential in-gel electrophoresis (2D-DIGE) combined with matrix-assisted laser desorption/ionization time of flight mass spectrometry (MALDI-TOF MS) to identify the changes in protein expression following *CD99* upregulation of L428 cells, and downregulation of m*CD99*L2 in A20 cells. We found that 21 proteins were upregulated and 17 downregulated in L428 cells with ectopic overexpression of *CD99*. Meanwhile, 21 upregulated proteins and 20 downregulated proteins were identified in A20 cells following silencing of m*CD99*L2 with siRNA. Among these identified proteins, *SEPTIN2* and *STATHMIN*, which mediate cytoskeletal reorganization and play an important role in cellular differentiation, were selected for further validation and functional analysis.

## Materials and Methods

### Cell culture and transfection

The cHL cell lines L428 (B-cell origin) [18, 19] and A20 (mouse B lymphoma cell line) were provided by Dr. Chan (the Nebraska Medical Center, Omaha, NE, USA). L428 cells were stably transfected with human *CD99* gene (L428-*CD99*) or negative control vector (L428-CTR), and A20 cells were stably transfected with shRNA targeting m*CD99* (A20-m*CD99*L2-) or negative control vector (A20-CTR) as previously described [13, 14, 20]. These cells were cultured in RPMI-1640 medium supplemented with 10% heat-inactivated fetal bovine serum (FBS) (Logan, UT, USA) at 37°C and 5% CO_2_.

### Fluorescent dye labeling, 2D-DIGE, and MALDI-TOF MS

The cells were lysed on ice in lysis buffer (Cell Signaling Technology, Boston, MA, USA) supplemented with protease and phosphatase inhibitor (Roche), and 1 mM PMSF (Sigma), and centrifuged at 15,000 × *g* for 30 min at 4°C. A total of 50 μg of protein was labeled with one of three CyDye DIGE Fluors (GE Healthcare). Protein samples from four different groups (L428-*CD99*, L428-CTR, A20-m*CD99*L2-, and A20-CTR) were labeled with Cy3 and Cy5, respectively. The internal standard contained equal amounts of each sample labeled with Cy2. CyDyes were combined with samples at a ratio of 400 pmol of CyDye to 50 μg of sample. The labeling reaction was performed on ice and in the dark for 30 min. The reaction was then quenched by incubating with 1.5 μL of 10 mM lysine on ice and in the dark for 10 min. Both groups of proteins (L428-*CD99* vs L428-CTR and A20-m*CD99*L2- vs A20-CTR) were pooled for 2-D gel electrophoresis for protein identification. 50 μg of each pooled protein sample was diluted in 450 mL of rehydration buffer for isoelectric focusing on Ettan IPGphor IEF System (GE Healthcare) following the manufacturer's instructions with IPG strips (24 cm, pH 3–10 NL, GE Healthcare). The IPG strips were rehydrated prior to use at 50 V for 10 h, followed by focusing at 100 V for 2 h, 300 V for 2 h, 600 V for 2 h, 1000 V for 2 h, 3000 V for 2 h, 9000 V for 2 h, and maintained at 500 V for a total of 90,000 V h. The two-dimensional separation was transferred onto 12% SDS-polyacrylamide gel electrophoresis (SDS-PAGE) on Ettan-DALT twelve perpendicular gel electrophoresis system (GE Healthcare). All CyDye-labeled gels were stained with Coomassie Blue R350 (GE Healthcare). The Cy2, Cy3, and Cy5-labeled images were scanned by a FLA5100 scanner system (GE Health) at the excitation/emission of 480 nm/530 nm (Cy2), 540 nm/590 nm (Cy3), and 620 nm/680 nm (Cy5).

Labeled image files were analyzed by DeCyder 2D 6.0 difference analysis software (GE Healthcare). All the protein spots from each gel were analyzed by Differential In-gel Analysis. The same protein spots on different gels were matched using an internal standard and analyzed using the DeCyder software (also known as BVA, Biological Variance Analysis). Differentially expressed protein spots of interest were excised from the stained gels and pooled together. The gel pieces were washed in 25 mM NH_4_HCO_3_, 50% acetonitrile (ACN) for 30 min, and then dehydrated in 100% ACN for 10 min. The proteins were digested using sequencing-grade trypsin (Promega Corporation) overnight at 37°C. Peptides were extracted in 5% TFA and 50% ACN, and dried using a Speedvac. The peptides were resuspended in 0.3% TFA, and co-crystallized by α-cyano-4-hydroxycinnamic acid (CHCA) matrix on a MALDI target. The proteins were identified using an ABI 4800 Proteomic Analyzer MALDI-TOF MS mass spectrometer (Applied Biosystems). Mass spectrometry spectra were identified in the Swiss Prot database using Global Proteome Server Explorer software (Applied Biosystems).

### SiRNA Transfection

Transfection of siRNA was carried out using Lipofectamine2000 Transfection Reagent (Invitrogen, CA) following the manufacturer’s protocols. For each transfection, 10 μg of siRNA oligos were used for 2 ×10^6^ cells. The siRNA sequences are listed in S1 Table. The transfection efficiency was determined by quantitative real-time PCR (qRT-PCR) in triplicate.

### RNA isolation, reverse transcription, and qRT-PCR analysis

Total RNA extraction, reverse transcription, and qRT-PCR were carried out as previously described [13]. Primers specific for human *SEPTIN2* and *STATHMIN* are indicated in S2 Table. Glyceraldehyde 3-phosphate dehydrogenase (GAPDH) was used as an internal control. The reaction conditions were 95°C for 30 sec, followed by 40 cycles of 95°C for 30 sec and 54°C for 34 sec. The relative mRNA levels were calculated using the 2^-△△Ct^ method. The qRT-PCR experiments were repeated independently three times.

### Western blot

Cells were harvested and washed twice with cold PBS. Cell lysates were prepared, and equal amounts of protein (50 μg) were separated on 8% SDS-PAGE, and transferred onto polyvinylidene difluoride (PVDF) membranes (Hercules, CA, USA). Membranes were incubated with 5% skim milk in TBS-0.1% Tween-20 for 2 h to block the residual binding sites followed by immunoblotting overnight at 4°C with appropriately diluted antibody. The antibodies used in this study are listed in S3 Table. Specific binding was revealed by mouse HRP-conjugated anti-rabbit IgG (Santa Cruz) and an enhanced chemiluminescence system (ECL-Plus; Amersham Biosciences, Piscataway, NJ, USA).

### Patients: sample selection and ethical statement

Formalin-fixed, paraffin-embedded archival specimens of cHL and reactive lymphoid hyperplasia (RH) were obtained from the Department of Pathology at the Nanfang Hospital affiliated to Southern Medical University from March 2009 to December 2013. All samples were reviewed and classified according to the World Health Organization criteria (2008). The study was scrutinized and approved by the Medical Ethics Committee of Southern Hospital of Southern Medical University. Written informed consent was obtained from each patient.

### Immunohistochemistry and immunocytochemistry analyses

Immunohistochemistry (IHC) and immunocytochemistry (ICC) analyses were performed as previously described [20]. The antibodies used are listed in S3 Table. Evaluation of the immunohistochemical staining results was conducted independently by two pathologists (T.Z. and XH.Z.) who were blinded to the clinical data. Staining was scored as positive if at least 10% of the tumor cells were immunoreactive, and then scored as weak (1+), moderate (2+), or strong (3+) according to staining intensity.

### Preparation of paraffin-embedded cell blocks

L428 cells (>5×10^7^/mL) were collected, washed twice with cold PBS, and then fixed in 10% formaldehyde overnight at room temperature without suspension. Next day, the cell block was packaged with a lens paper and placed in the paraffin-embedded box, followed by IHC.

### Immunofluorescence

Cells (2.0×10^5^/ml) were inoculated into each well of 6-well plates (Corning, NY, USA) and cultured in complete medium for 48 h followed by in serum-free medium for another 24 h. After deposition, fixation and permeabilization, the cells were labeled with appropriate antibodies. The antibodies used are listed in S3 Table. Negative controls were performed by replacing the primary antibodies with PBS. The cells were observed under a fluorescence microscope (Tokyo, Japan).

### Flow cytometry

Control L428 cells and L428 cells transfected with *STATHMIN*-siRNA for 96h were harvested, washed twice with PBS and suspended at a concentration of 1×10^7^/mL. Cell surface expression of CD15, CD45, CD38 and CD138 were analyzed by FC 500 MPL flow cytometer (CA). The antibodies applied are listed in S4 Table. Data were analyzed in FCS Express V3 software (DeNovo Software, Canada).

### Statistical analysis

All images, including Western blots and flow cytometry, were representative of at least three independent experiments. Quantitative RT-PCR assays were carried out in triplicate for each experiment. The data shown are expressed as the mean ± SD. Statistical analysis was performed using SPSS version 13.0 software. All comparisons were paired two-sample Student’s t-tests, except that qRT-PCR experiments with *STATHMIN*-siRNAs or *SEPTIN2*-siRNAs were One-way ANOVA. P-value of <0.05 was considered statistically significant. Proteomic database analysis to identify the proteins was based on peptide mass fingerprinting (PMF) data by searching MASCOT (http://www.matrixscience.com) software using a MASCOT Distiller, which detects peaks by fitting an ideal isotopic distribution to the experimental data. The parameters were set as follows: mass tolerance 250 ppm, number of missed cleavage sites allowed 1, cysteine residue modified as carbamidomethylcys, variable oxidative modifications (M), minimum number of matched-peptides 5, species selected as *Homo sapiens* (human) or *Mus musculus* (mice), with monoisotopic mass values. The mass and protein scores were obtained by searching MASCOT.

## Results

### Proteomic analysis: differential expression of proteins

**Fig 1:**
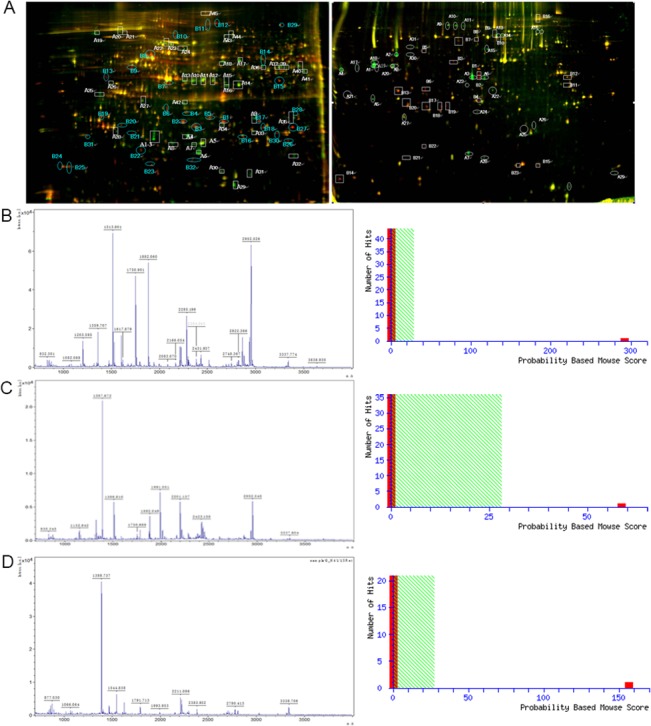
Representative 2D-DIGE, PMF and Mascot score maps of L428 cells and A20 cells. **(**A**)** Left panel: Protein spots expressed differentially between L428-*CD99* cells and L428-CTR cells were marked on the maps and further identified by MALDI-TOF MS. Right panel: Protein spots expressed differentially between A20-m*CD99*L2- cells and A20-CTR cells were marked on the maps and further identified by MALDI-TOF MS. **(**B**)** Left panel: MALDI-TOF mass spectrum of spot B15 obtained from L428 cells (spot B15 corresponds to *SEPTIN2*) after trypsin digestion. Right panel: The Mascot score of spot B15 confirmed by MALDI-TOF MS was 292**. (**C**)** Left panel: MALDI-TOF mass spectrum of spot A8 obtained from A20 cells (spot A8 corresponds to *SEPTIN2*) after trypsin digestion. Right panel: The Mascot score of spot A8 confirmed by MALDI-TOF MS was 58. **(**D**)** Left panel: MALDI-TOF mass spectrum of spot A28 obtained from A20 cells (spot A28 corresponds to *STATHMIN*) after trypsin digestion. Right panel: The Mascot score of spot A28 confirmed by MALDI-TOF MS was 156.

We previously found that upregulation of *CD99* in H/RS cells induced terminal B-cell differentiation [13], while downregulation of m*CD99*L2 in BALB/c mice led to the transformation of A20 cells into H/RS-like cells [14]. To explore the mechanism underlying the *CD99*-mediated differentiation of H/RS cells, we conducted differential proteome analyses of the L428-*CD99* cells vs L428-CTR cells using 2D-DIGE with MALDI-TOF MS. A number of spots were found to be significantly altered (P < 0.05) after upregulation of *CD99* (Fig 1 and S1 Fig). Thirty-eight single spots were successfully identified using PMF (S5 Table). Of the 38 spots, 21 were upregulated and 17 were downregulated in L428-*CD99* cells compared with L428-CTR cells. To determine the potential functions of the differentially expressed proteins, we performed protein congregation analysis based on the Gene Ontology (GO) software suite (http://61.50.138.115/GOfact/) in the terms of cellular component, biological process and molecular function (S2 Fig), and identified several closely related proteins that were potentially involved in *CD99*-mediated cell differentiation (S6 Table).

We also compared the protein expression profile of A20-m*CD99*L2- cells vs A20-CTR cells using the same methods. Results of 2D-DIGE, PMF, and Mascot-score maps are illustrated in Fig 1 and S1 Fig. Forty-one differentially expressed proteins were identified by PMF. Of these, 21 were upregulated and 20 were downregulated in the A20-m*CD99*L2- cells compared to the A20-CTR cells (S7 Table). Protein congregation analysis with Gene Ontology identified several proteins closely related to m*CD99*L2-mediated cell differentiation (S8 Table).

*SEPTIN2*, associated with cytoskeletal organization, was identified to be significantly downregulated in L428-*CD99* cells (Fig 1B and S5 Table) but upregulated in A20-m*CD99*L2- cells (Fig 1C and S7 Table). In addition, *STATHMIN* was also overexpressed in A20-m*CD99*L2- cells (Fig 1D and S7 Table). It is well documented that both *SEPTIN2* and *STATHMIN* regulate cytoskeletal organization [21, 22], which is associated with cellular differentiation [6]. Accordingly, we selected these two proteins for further investigation.

### Differential expression of *SEPTIN2* and *STATHMIN* in L428-CD99 and L428-CTR cells

**Fig 2:**
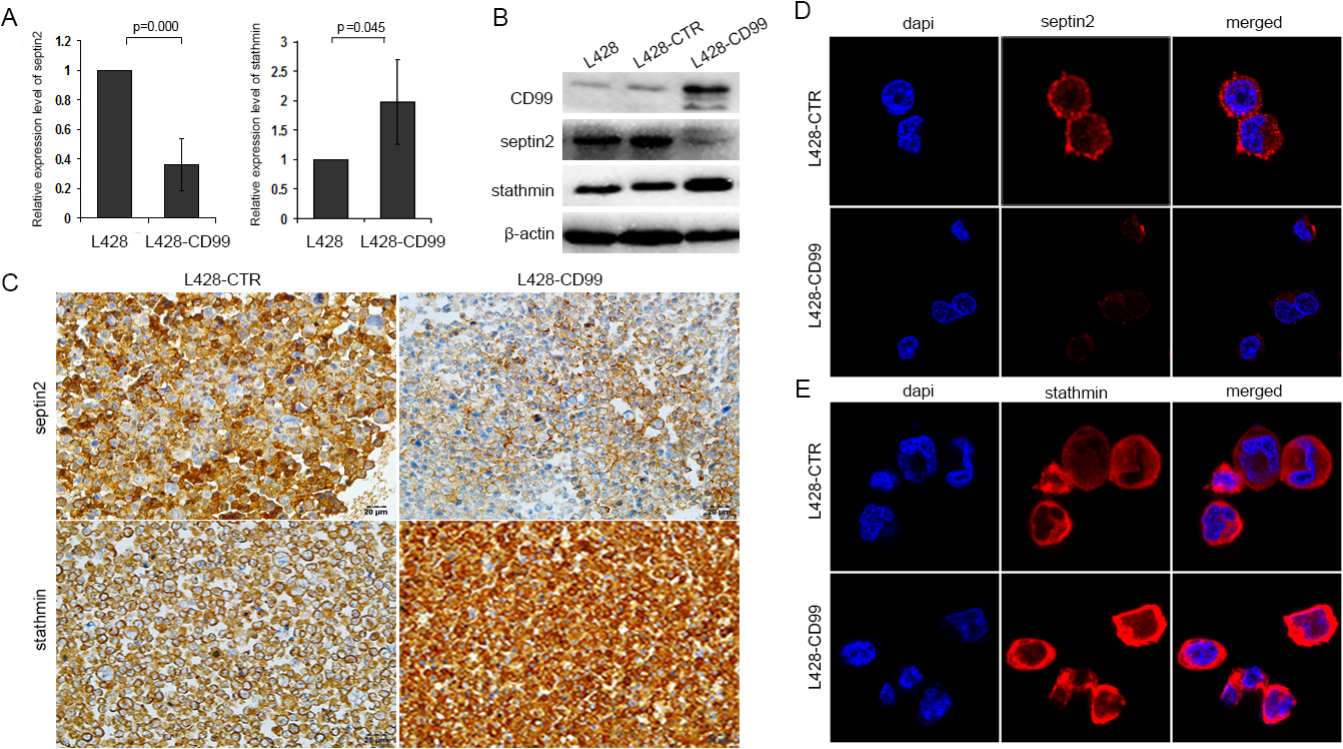
Expression of *SEPTIN2* and *STATHMIN* in L428-*CD99* and L428-CTR cells. Expression levels of *SEPTIN2* and *STATHMIN* were detected by qRT-PCR (A), Western blot (B), ICC (C), and Confocal microscopy (D, E) (original magnification ×400).

We next validated the expression status of *SEPTIN2* and *STATHMIN* in L428-*CD99* and L428-CTR cells by qRT-PCR, Western blot, ICC, and immunofluorescence. Compared with L428-CTR cells, both the mRNA (Fig 2A) and protein (Fig 2B) levels of *SEPTIN2* were significantly decreased, while those of *STATHMIN* were increased in L428-*CD99* cells. Similar downregulation of *SEPTIN2* and upregulaton of *STATHMIN* in L428-*CD99* cells were revealed by ICC (Fig 2C) and immunofluorescence (Fig 2D). In addition, ICC and immunofluorescence images showed that *SEPTIN2* and *STATHMIN* were primarily found in the cytoplasm (Fig 2E). These results showed that *SEPTIN2* expression was negatively correlated with *CD99*, whereas a synergistic expression between *CD99* and *STATHMIN* was observed, which was further validated in the Burkitt’s lymphoma cell line BJAB following silencing of *CD99* by siRNA (S1 Text).

### Differential expression of *SEPTIN2* and *STATHMIN* in cHL and RH tissues

**Fig 3:**
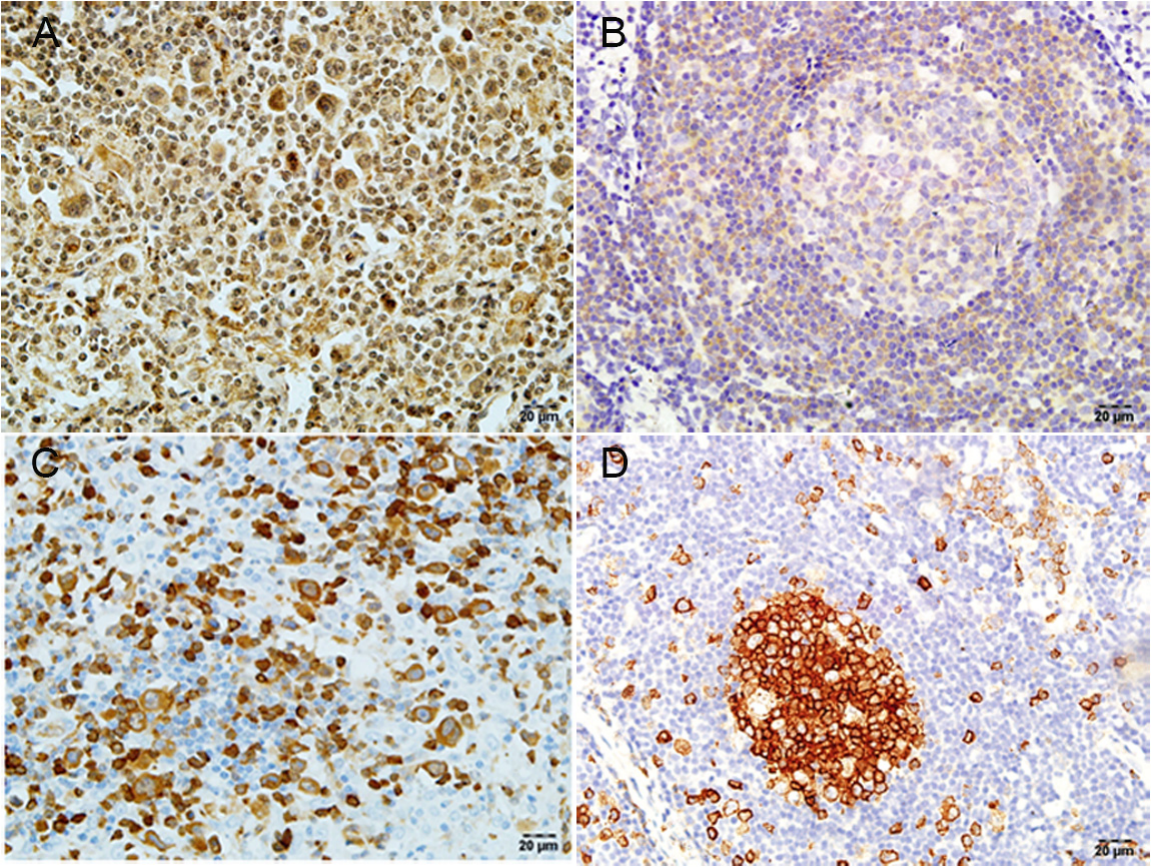
Detection of *SEPTIN2* and *STATHMIN* by IHC in cHL and RH tissues. (A) Expression of *SEPTIN2* in cHL. (B) Expression of *SEPTIN2* in RH. (C) Expression of *STATHMIN* in cHL. (D) Expression of *STATHMIN* in RH. Original magnification ×400. Positive staining appears reddish brown.

The foregoing results suggest that upregulation of *CD99* leads to decreased *SEPTIN2* and increased *STATHMIN* levels in L428 cells. Therefore, we examined the expression of *SEPTIN2* and *STATHMIN* in cHL (n = 20) and RH (n = 5) tissues by IHC. Representative examples of positive immunostaining are shown in Fig 3. Strong positive staining for *SEPTIN2* and *STATHMIN* was detected in 4/20 and 8/20 cHL samples, respectively (S9 Table). In RH tissues, strong *SEPTIN2* staining was not detected in any of the 5 cases; however, moderately positive staining was detected in 4/5 (80%) and weakly positive was found in 1/5 (20%). Strong *STATHMIN* staining was detected in 3/5 cases (60%), with the remainder (40%) showing moderate to intermediate staining.

Moreover, in RH tissues, there was strong staining for *STATHMIN* in the GC, and less dense, more scattered staining in the mantle zones. Unlike *STATHMIN*, *SEPTIN2* showed weak staining in the GC, but stronger expression in the mantle zones. In capp:ds:hodgkin lymphoma(hl)HL tissues, histologically-confirmed H/RS cells showed strong to intermediate *STATHMIN* expression, and variable background *STATHMIN* expression. In contrast, *SEPTIN2* showed intermediate to strong expression in the cytoplasm of H/RS cells, but slightly stronger expression in background Non-H/RS cells. We also observed stronger staining for *STATHMIN* in the RH cases than in the cHapp:ds:hodgkin lymphoma(hl)L, but weaker expression of *SEPTIN2*.

### SiRNA silencing of *SEPTIN2* results in cytoskeletal reorganization

**Fig 4:**
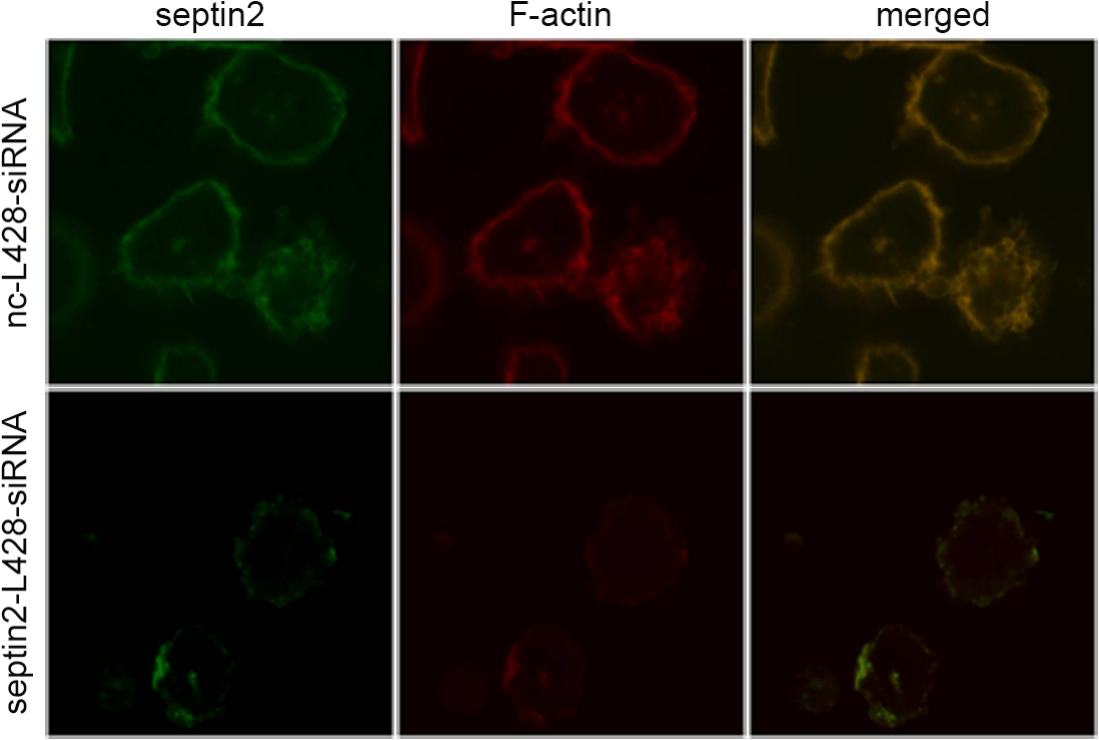
Transfection of *SEPTIN2* siRNA resulted in cytoskeleton change in L428 cells. The Rhodamine-phalloidin was applied for visualizing *F-actin*. The overlay shows co-localization in yellow.

Bioinformatics analysis indicated that *SEPTIN2* was associated with cytoskeletal organization (S6 and S8 Tables). The differential expression of *SEPTIN2* in L428-CTR and L428-*CD99* cells suggests that *SEPTIN2* might play a role in the regulation of the cytoskeletal dynamics in cHL. To test this hypothesis, we silenced *SEPTIN2* with siRNA and visualized *SEPTIN2* and *F-actin* in L428 cells by immunofluorescence. As shown in Fig 4, *SEPTIN2* colocalized with *F-actin* in the cytoplasm of the control cells with a punctate distribution at the cell-substratum interface. SiRNA-mediated downregulation of *SEPTIN2* reduced the expression of *SEPTIN2* and *F-actin* in L428 cells. Moreover, *SEPTIN2* downregulation led to fewer filopodia and thinner cortical filamentous actin at the cell surface. Thus, downregulation of *SEPTIN2* may induce cytoskeletal rearrangement in L428 cells. The findings were consistent with our previous study showing that *CD99*-upregulated L428 cells underwent terminal B-cell differentiation and displayed cytoskeleton reorganization, disappearance of filopodia and thinning of cortical filamentous actin [13]. The *CD99*-upregulated L428 cells showed reduced expression of *SEPTIN2*. Taken together, these results suggest that *SEPTIN2* plays a role in maintaining H/RS cell morphology and *SEPTIN2*-induced cytoskeletal reorganization may contribute to *CD99*-mediated differentiation of H/RS cells.

### Dynamic expression of *STATHMIN* is correlated with B-cell lymphoma differentiation

**Fig 5:**
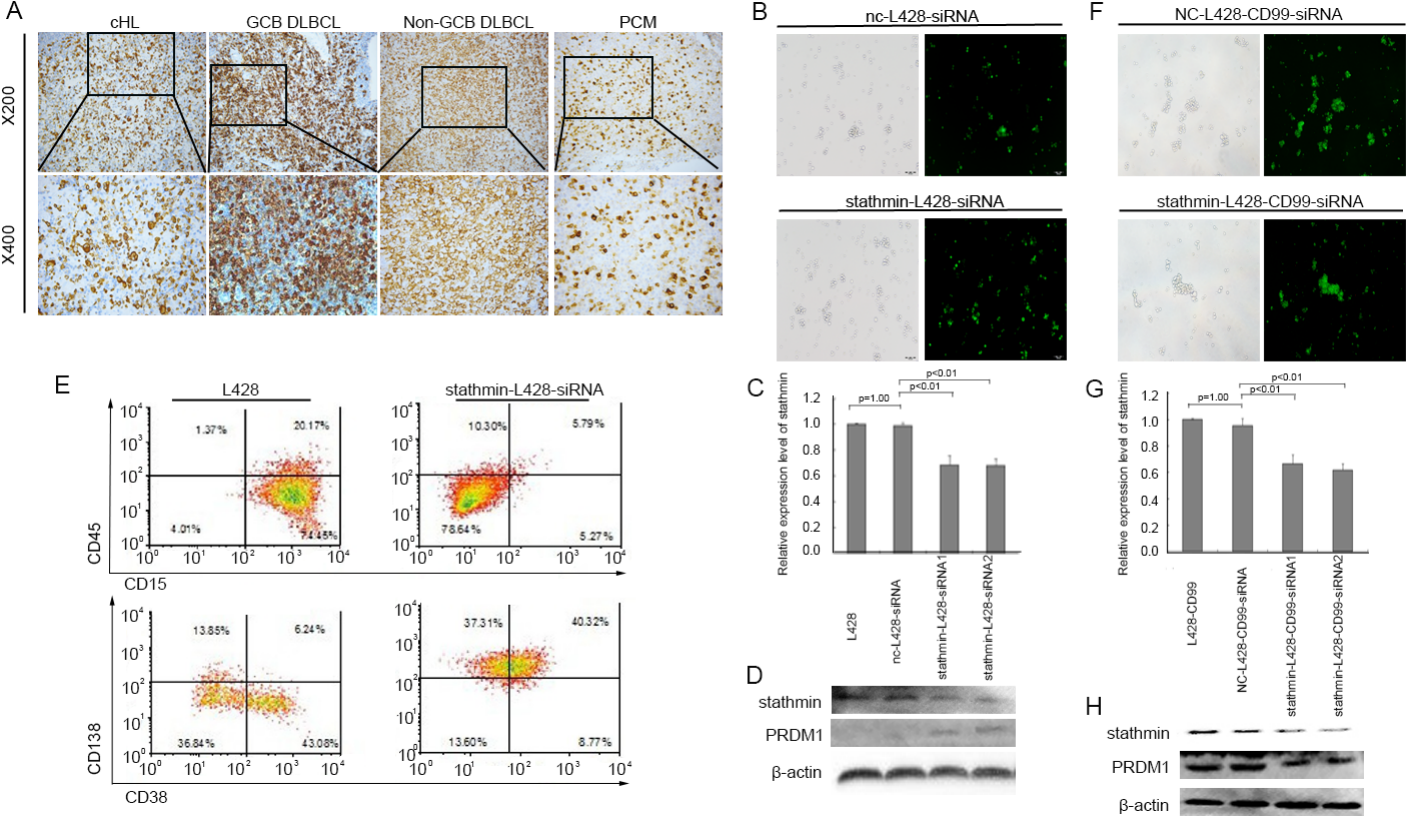
*STATHMIN* was involved in the differentiation of B-cells. (A) IHC analysis of *STATHMIN* expression in different types of lymphomas (cHL, GCB-DLBCL, Non-GCB DLBCL, and PCM) (B) Morphological images of L428 cells transfected with *STATHMIN*- siRNA. Left panel: light image, Right panel: fluorescent image. Original magnification ×100. (C) Relative expression level of *STATHMIN* in L428 transfected with *STATHMIN*-siRNA for 24h by qRT-PCR. (D) Western blot of *PRDM1* expression in L428 cells transfected with *STATHMIN*-siRNA for 96h. (E) Flow cytometry analysis of the surface expression of B-cell differentiation antigens in L428 cells transfected with *STATHMIN*-siRNA for 96h. (F) Morphological images of L428-*CD99* cells transfected with *STATHMIN*-siRNA. Left panel: light image, Right panel: fluorescent image. Original magnification ×100. (G) Relative expression levels of *STATHMIN* in L428-*CD99* transfected with *STATHMIN*-siRNA for 24h by qRT-PCR. (H) Western blot of *PRDM1* expression in L428-*CD99* cells transfected with *STATHMIN*-siRNA for 96h.

*STATHMIN* plays an important role in cellular differentiation [22–24]. We previously showed that overexpression of *CD99* promoted the differentiation of lymphoma cells into terminal B-cells [13]. The differential expression of *STATHMIN* in L428-CTR and L428-*CD99* cells suggests that *STATHMIN* may contribute to the differentiation of HL. To test this, we evaluated *STATHMIN* expression by IHC in 65 lymphoid neoplasms, including 20 cHL (S10 Table). In RH, the majority of the *STATHMIN*-positive cells were located in the GC, and only occasionally found in the interfollicular areas. In cHL, strong to intermediate staining of *STATHMIN* was detected in H/RS cells. Increasingly variable expression levels were found in other lymphoid cells. DLBCL were also stained positive for *STATHMIN* with varying degrees of intensity. The staining intensity of *STATHMIN* in DLBCL derived from GCB cells and plasmacytomas (PCM) was stronger than that in the non-GCB cells (Fig 5A). These data demonstrate that *STATHMIN* expression decreases from the GC to post-GC stages, and increases from the post-GC to plasma stages. Our results were consistent with the previous finding that *STATHMIN* was involved in B-cell lymphoma differentiation [25].

### Downregulation of *STATHMIN* induces H/RS cell differentiation toward plasma cells

To further confirm the role of *STATHMIN* in HL differentiation, we downregulated *STATHMIN* by siRNA in L428 cells (Fig 5B and 5C) and assessed the expression of *PRDM1*, which plays an important role in terminal differentiation of B-cells into immunoglobulin (Ig)-secreting plasmablasts and plasma cells [26]. SiRNA-mediated *STATHMIN* downregulation led to increase in *PDRM1* protein level (Fig 5D). We next evaluated the expression of B-cell differentiation markers using flow cytometry. As shown in Fig 5E, the percentage of cells expressing CD15, the diagnostic marker for cHL, was obviously reduced in *STATHMIN*-L428-siRNA cells compared with the control cells. Consistent with the role for *STATHMIN* in B-cell differentiation, plasma cell phenotypic markers CD38 and CD138 were increased following *STATHMIN* silencing. We also silenced *STATHMIN* in L428-*CD99* cells (Fig 5F and 5G). In sharp contrast to L428-*CD99* cells, *PRDM1* protein level was reduced after silencing of *STATHMIN* in L428-*CD99* cells (Fig 5H). Taken together, these results suggest *STATHMIN* induced differentiation of H/RS cells to plasma cells.

### *SEPTIN2* silencing leads to downregulation of *STATHMIN*

**Fig 6:**
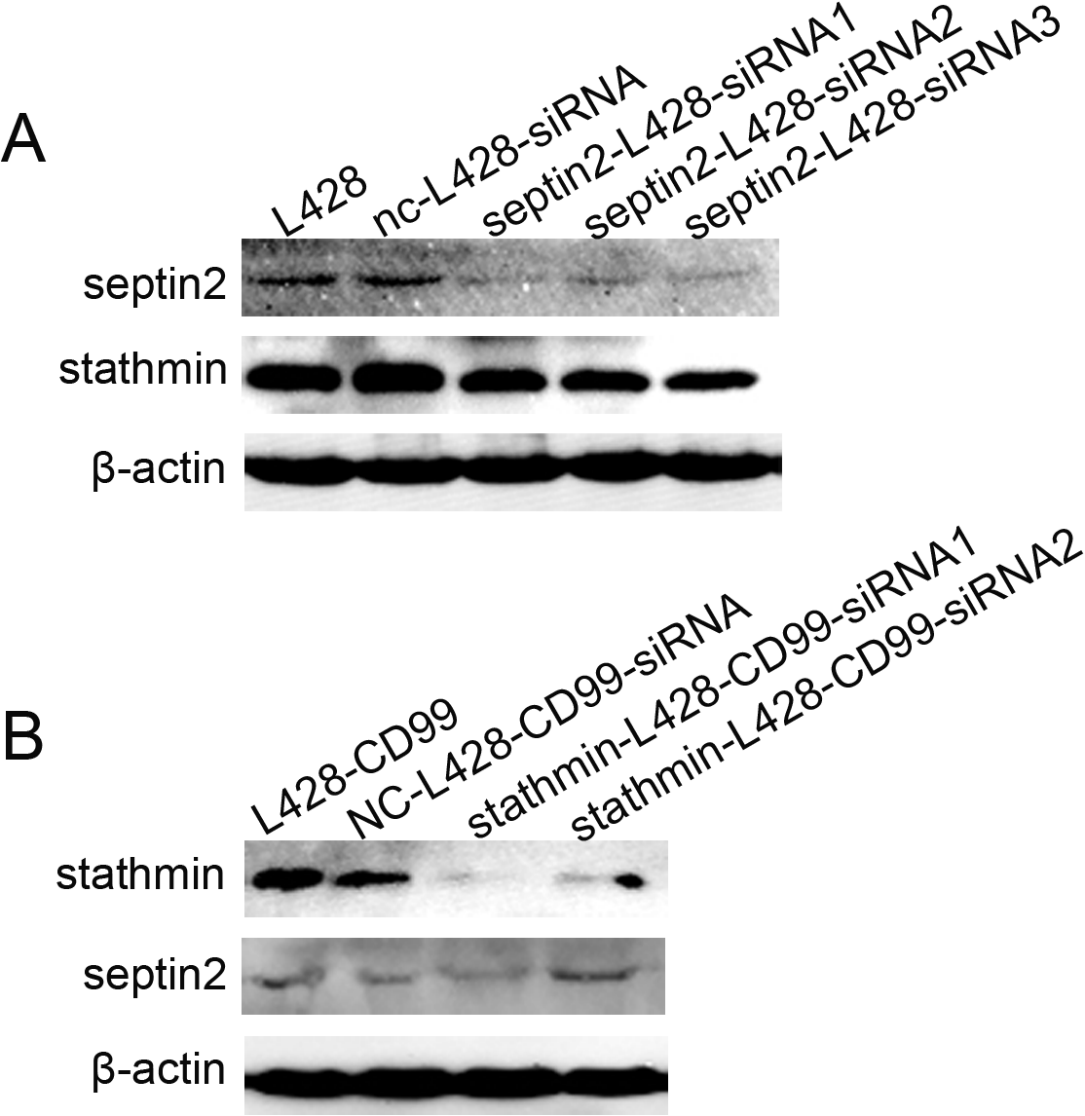
Correlation between *SEPTIN2* and *STATHMIN* protein expression. (A) Protein expression levels of *SEPTIN2* and *STATHMIN* in L428 cells transfected with *SEPTIN2*-siRNAs. (B) Protein expression levels of *SEPTIN2* and *STATHMIN* in L428-*CD99* cells transfected with *STATHMIN*-siRNAs.

Our results clearly showed that *SEPTIN2* and *STATHMIN* were closely associated with *CD99* expression, suggesting a potential relationship between *SEPTIN2* and *STATHMIN*. Therefore, we examined the expression of both *SEPTIN2* and *STATHMIN* in L428 cells after downregulation of *SEPTIN2* with siRNA and in L428-*CD99* cells following downregulation of *STATHMIN* with siRNA, respectively. Surprisingly, *STATHMIN* expression was reduced when the expression of *SEPTIN2* was decreased in L428 cells (Fig 6A). In contrast, almost no change in *SEPTIN2* levels was observed in the L428-*CD99* cells following *STATHMIN* downregulation (Fig 6B). These results indicate that *SEPTIN2* regulates *STATHMIN*. The mechanism remains to be determined.

## Discussion

In this study, we elucidated the molecular mechanisms of *CD99*-mediated H/RS cellular differentiation, using 2D-DIGE and MALDI-TOF MS analyses and identified proteins that were differentially expressed in L428-*CD99* vs L428-CTR cells and A20-m*CD99-*L2- vs A20-CTR cells. *SEPTIN2* and *STATHMIN*, which are involved in cytoskeleton organization, were found to be significantly expressed (S6 and S8 Tables), and were selected for further validation and functional analysis.

*SEPTIN2* is a member of the septin family that is involved in many cellular functions, such as cell polarity, cell cortex compartmentalization, vesicle transport, and regulation of the actin and tubulin cytoskeleton [27]. Septins are highly expressed in many tumors [28]. Moreover, septins are also associated with a filament-forming cytoskeletal GTPase, which is required for normal organization of the actin cytoskeleton [29]. Actin cytoskeleton not only plays a pivotal role in the regulation of the morphology and apoptosis of B-cells [30], but also mediates the BCR signal transmission during B-cell differentiation [31]. Depolymerization of the actin cytoskeleton inhibits surface BCR clustering [32] and induces a signaling cascade in the absence of antigen [33]. In addition, evidence suggests that *SEPTINS* play an important role in cytoskeletal dynamics. *SEPTIN*-*F-actin* linkage may contribute to higher-order organization of the cortical cytoskeleton [34, 35]. The relationship between *SEPTIN* filaments and actin has been well established, however, very little is known about its role in HL and H/RS cell differentiation. In this study, we found that *SEPTIN2* expression was negatively correlated with *CD99* and *SEPTIN2* downregulation triggered the reduction of *F-actin* and pseudopodia apophysis in L428 cells. These results were consistent with the previous finding that upregulation of *CD99* altered cytoskeleton in L428 cells [13]. These data indicate that *SEPTIN2* plays an important role in maintaining H/RS cell shape. Thus, *SEPTIN2*-mediated cytoskeleton reorganization may be one of the induction mechanisms of H/RS cellular differentiation by *CD99* overexpression. Nevertheless, the relationship between *SEPTIN2* and BCR is unknown and needs further investigation.

*STATHMIN* is another cytoskeletal protein, which is overexpressed in several malignancies with a significant role in cell differentiation [23]. Reducing the level of *STATHMIN* reverses many phenotypes associated with transformation [36]. Moreover, *STATHMIN* is a highly conserved cytosolic phosphoprotein implicated in regulating microtubule dynamics [37, 38], cell proliferation and differentiation [39–42]. Nylander et al. reported that *STATHMIN* exhibited variable expression in malignant lymphomas and proposed that it may be involved in B-cell differentiation [25]. Our results obtained from IHC support this idea. However, whether *STATHMIN* participates in *CD99*-mediated H/RS cell differentiation toward terminal B-cells is unclear.

Interestingly, the present study demonstrated that downregulation of *STATHMIN* in L428 cells resulted in reduction of CD15, a characteristic marker of H/RS cells [43], and expression of plasma-cell markers CD38 and CD138. CD38 expression is tightly regulated during B-cell ontogenesis and is present at high levels in terminally differentiated plasma cells [44]. Furthermore, *PRDM1* is a necessary and crucial factor in the regulation of B-cell differentiation toward plasma cells [26]. The results showed that silencing of *STATHMIN* increased the expression of *PRDM1* in L428 cells. Our results suggested that downregulation of *STATHMIN* may stimulate L428 cellular differentiation toward plasmablasts or plasma cells (terminal B-cells). In addition, we investigated the role of *STATHMIN*-mediated differentiation in *CD99* cells, by silencing *STATHMIN* in L428-*CD99* cells with siRNA. We found that silencing of *STATHMIN* led to decrease in *PRDM1*, which is in contrast to that in L428 cells. Therefore, it is possible that *STATHMIN* was involved in the regulation of *CD99*-mediated differentiation of H/RS cells toward terminal B-cells.

In addition, our previous study demonstrated that ectopic overexpression of *CD99* resulted in cell growth inhibition in L428 cells, but the underling mechanism was unclear [13]. It was reported that *STATHMIN* regulates dynamics of the mitotic spindle by promoting microtubule depolymerization [45]. When cells enter mitosis, phosphorylation-dependent inactivation of *STATHMIN* allows microtubules to polymerize and assemble into a mitotic spindle. The microtubule-depolymerizing activity of *STATHMIN* must be restored in the later phases of mitosis to allow mitotic spindle disassembly and proper exit from mitosis [37]. Both overexpression and downregulation of *STATHMIN* results in mitotic spindle abnormalities and accumulation of cells in G2/M phases of the cell cycle [46]. Therefore, we speculate that downregulation of *STATHMIN* in L428-*CD99* cells may contribute to its growth inhibition.

To the best of our knowledge, we are the first to report the expression of *SEPTIN2* in cHL tissues. The staining intensity of *SEPTIN2* in the cytoplasm of H/RS cells was weaker than in other inflammatory cells; whereas in RH tissues, *SEPTIN2* was expressed at higher levels in the mantle zone than in the GC. These results suggest that the patterns of *SEPTIN2* expression in RH and cHL were contrary to *STATHMIN* expression, for unknown reasons.

Furthermore, several proteins known to be involved in cHL like *ARHGDIB*, *MSH2* or *PRDX2* were found differentially expressed in L428 cells as well as in m*CD99*L2 downregulated A20 cells (S5 and S7 Tables). Evidence shows that the guanosine triphosphatase (GTPase) inhibitor *ARHGDIB* is downregulated in H/RS cells and the absence of *ARHGDIB* might contribute to the apoptotic resistance of H/RS [47]. *PRDX2* is also downregulated in H/RS cells and epigenetic silencing of this gene may contribute to the loss of B-cell identity and survival of H/RS cells [48]. *MSH2* transcript is present in most B-cell lymphoma with the exception of plasma cell lymphoma and deregulation of *MSH2* in B-cell lymphoma types is characterized by aggressive biologic behavior [49]. In this study, proteomic analysis showed upregulation of *CD99* in L428 cells led to high expression of *ARHGDIB* and *PRDX2*, while low expression of *MSH2*. These results suggest that *ARHGDIB*, *PRDX2* and *MSH2* may play a role in *CD99*-induced transformation of H/RS cells toward B-cell.

**Fig 7:**
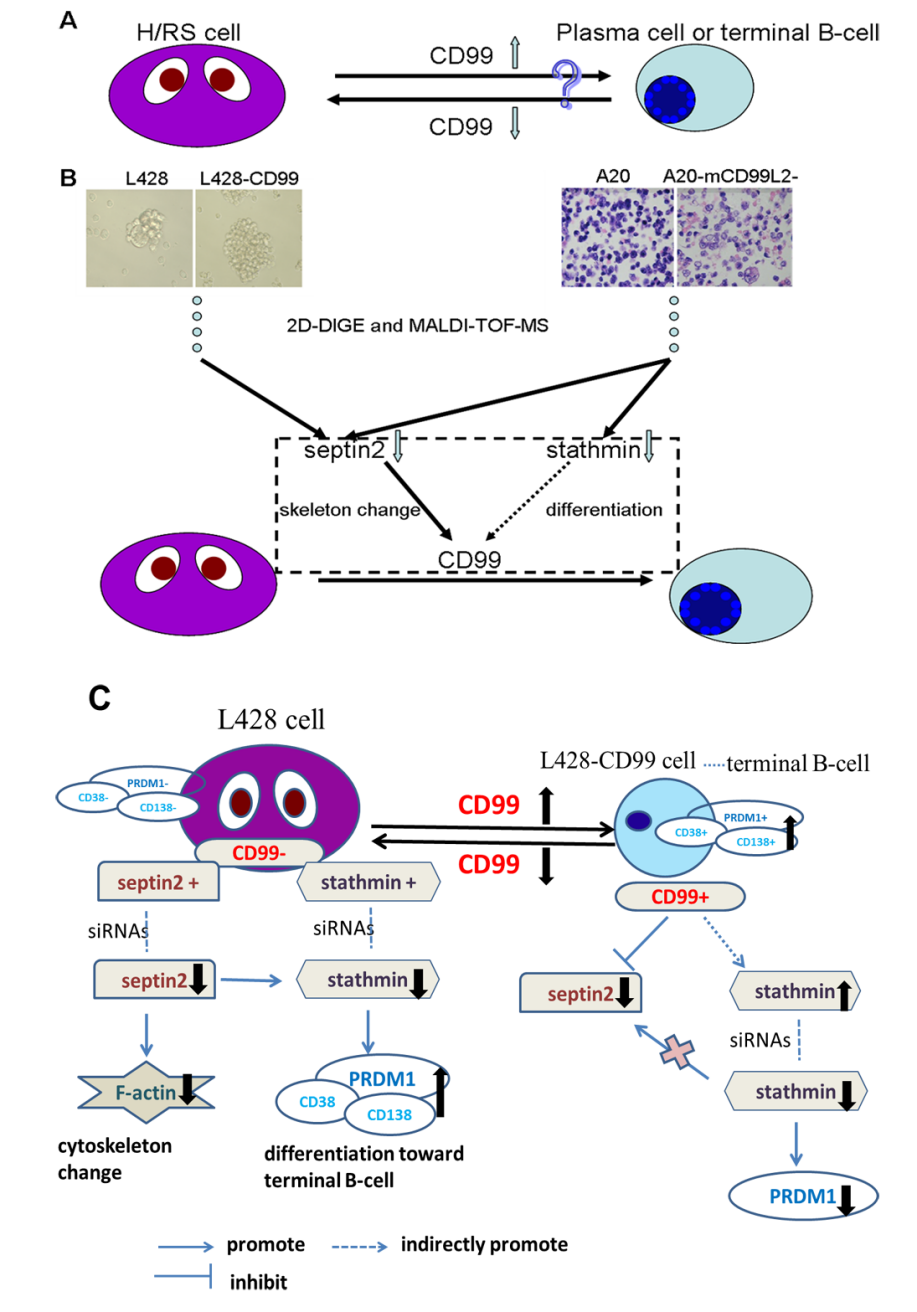
Summary figure. (A) *CD99* downregulation leads to the transformation of murine B lymphoma cells (A20) into cells with a H/RS phenotype, whereas *CD99* upregulation induces differentiation of classical Hodgkin’s lymphoma (cHL) cells (L428) into terminal B-cells. (B) 2D-DIGE and MALDI-TOF MS identified differentially expressed proteins respectively in *CD99* upregulation (L428-*CD99)* vs mock L428 cells, and in mouse *CD99* antigen-like 2 (m*CD99*L2) downregulation (A20-m*CD99*L2-) vs mock A20 cells. *SEPTIN2* and *STATHMIN* were chosen for further study. We found that *SEPTIN2* induced the cellular cytoskeleton reorganization in L428 cells and downregulation of *STATHMIN* induced L428 cells differentiation toward terminal B-cells, which partially explained the observation that upregulation of *CD99* induced H/RS cells to differentiate toward terminal B-cell. (C) Schematic model of the regulation of genes expression in L428 and L428-*CD99* cells. Low expression of *CD99* and high expression of *SEPTIN2* and *STATHMIN* were present in L428 cells. Downregulation of *SEPTIN2* with siRNAs in L428 cells induced change of *F-actin* expression. Downregulation of *STATHMIN* with siRNAs in L428 cells increased the expression of *PRDM1*, CD38 and CD138, which suggests the treated cell were differentiated toward terminal B-cell. Upregulation of *CD99* in L428 cells decreased *SEPTIN2* while increased *STATHMIN*. Downregulation of *STATHMIN* in L428-*CD99* cells with siRNAs reduced the expression of *PRDM1*.

In summary, we characterized the expression pattern of *SEPTIN2* in cHL and provided evidence that *SEPTIN2*-mediated cytoskeleton reorganization plays an important role in H/RS cell differentiation. Furthermore, we found that *CD99* induced the transformation of H/RS cells toward B-cell by regulating the expression of *SEPTIN2* and *STATHMIN* (Fig 7). The present study provides novel insights into the mechanisms underlying *CD99*-mediated H/RS cell differentiation toward B-cells.

## Supporting Information

S1 Fig Representative DIGE fluorescence images. (A-D) Representative DIGE images of L428-*CD99* and L428-CTR cells. (A) Cy3-labeled images of L428-*CD99* cells. (B) Cy5-labeled images of L428-CTR cells. (C) Internal images labeled with Cy2. (D) Merged images of the Cydye-labeled images. (E-H) Representative DIGE images of A20-m*CD99*L2- and A20-CTR cells. (E) Cy3-labeled images of A20-m*CD99*L2- cells. (F) Cy5-labeled images of A20-CTR cells. (G) Internal images labeled by Cy2. (H) Merged images of the Cydye-labeled images.
(TIF)

S2 Fig Gene ontology analysis. (A) Biological process annotation. (B) Cellular component annotation. (C) Molecular function annotation.
(TIF)

S3 Fig Relative expression level of *STATHMIN* when L428 or L428-*CD99* cells were treated with *STATHMIN*-siRNA for 72h. Left panel: relative expression level of *STATHMIN* in L428 cells transfected with *STATHMIN*-siRNA for 72h by qRT-PCR. Right panel: relative expression levels of *STATHMIN* in L428-*CD99* cells transfected with *STATHMIN*-siRNA for 72h by qRT-PCR.
(TIF)

S1 Table SiRNA sequences used in transfection experiments. (XLSX)

S2 Table Primers used in qRT-PCR analysis. (XLSX)

S3 Table Antibodies used for IHC, ICC, western blot, immunofluorescence analysis. (XLSX)

S4 Table Antibodies used for flow cytometry analysis. (XLSX)

S5 Table MALDI-TOF MS identification of 38 characteristic proteins with differential expression in L428-*CD99* vs L428-CTR cells. (XLSX)

S6 Table Closely related proteins involved in the *CD99* regulation of cell transformation. (XLSX)

S7 Table MALDI-TOF MS identification of 41 characteristic proteins with differential expression levels in A20-m*CD99*L2- vs A20-CTR cells. (XLSX)

S8 Table Closely related proteins involved in the m*CD99*L2 regulation of cell transformation. (XLSX)

S9 Table IHC analyses of *SEPTIN2* and *STATHMIN* in cHL and RH. (XLSX)

S10 Table IHC analyses of *STATHMIN* in lymphoma subtypes. (XLSX)

S1 Text Relationship between *CD99* and *STATHMIN* was tested in the L428-*CD99* cells and BJAB cells. (DOC)
