# Supplementary figures and images for: SEPTIN2 and STATHMIN Regulate CD99-Mediated Cellular Differentiation in Hodgkin's Lymphoma
Source: PLoS One. 2015 May 22;10(5):e0127568. doi: 10.1371/journal.pone.0127568 (PMC4441373; doi:10.1371/journal.pone.0127568)

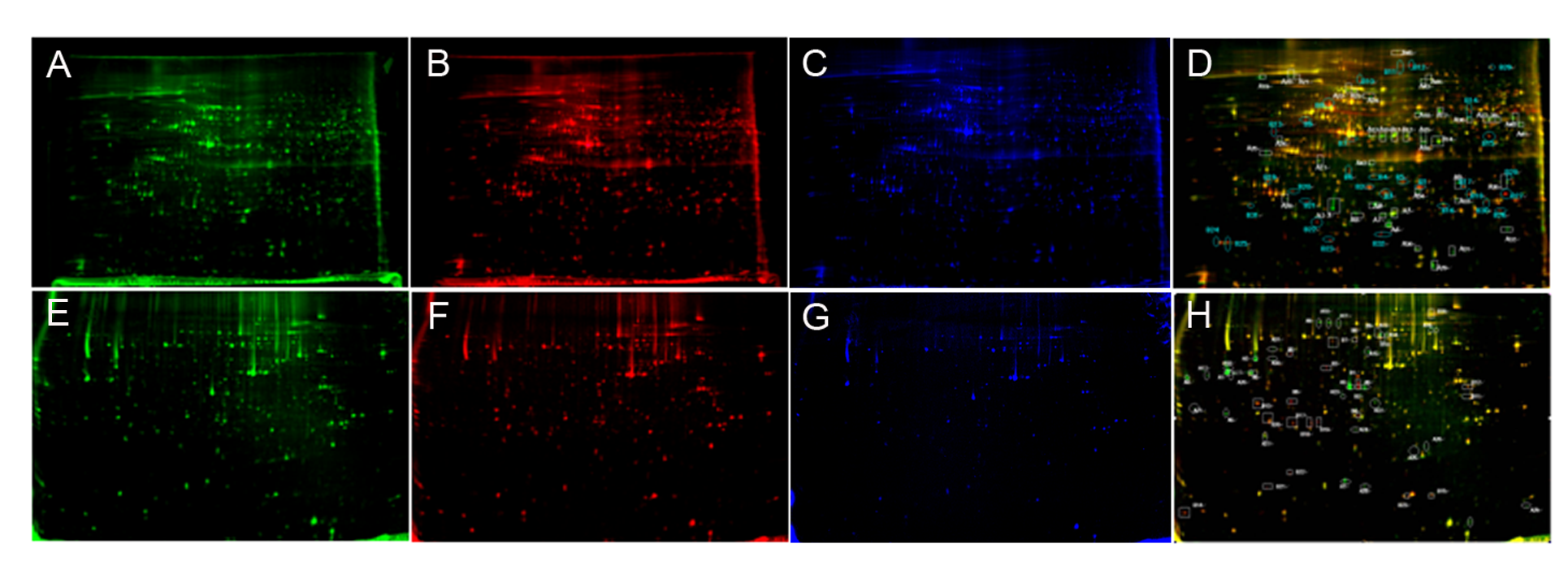

Supplement: S1 Fig — (A-D) Representative DIGE images of L428-CD99 and L428-CTR cells. (A) Cy3-labeled images of L428-CD99 cells. (B) Cy5-labeled images of L428-CTR cells. (C) Internal images labeled with Cy2. (D) Merged images of the Cydye-labeled images. (E-H) Representative DIGE images of A20-mCD99L2- and A20-CTR cells. (E) Cy3-labeled images of A20-mCD99L2- cells. (F) Cy5-labeled images of A20-CTR cells. (G) Internal images labeled by Cy2. (H) Merged images of the Cydye-labeled images. (TIF) [file pone.0127568.s001.tif]

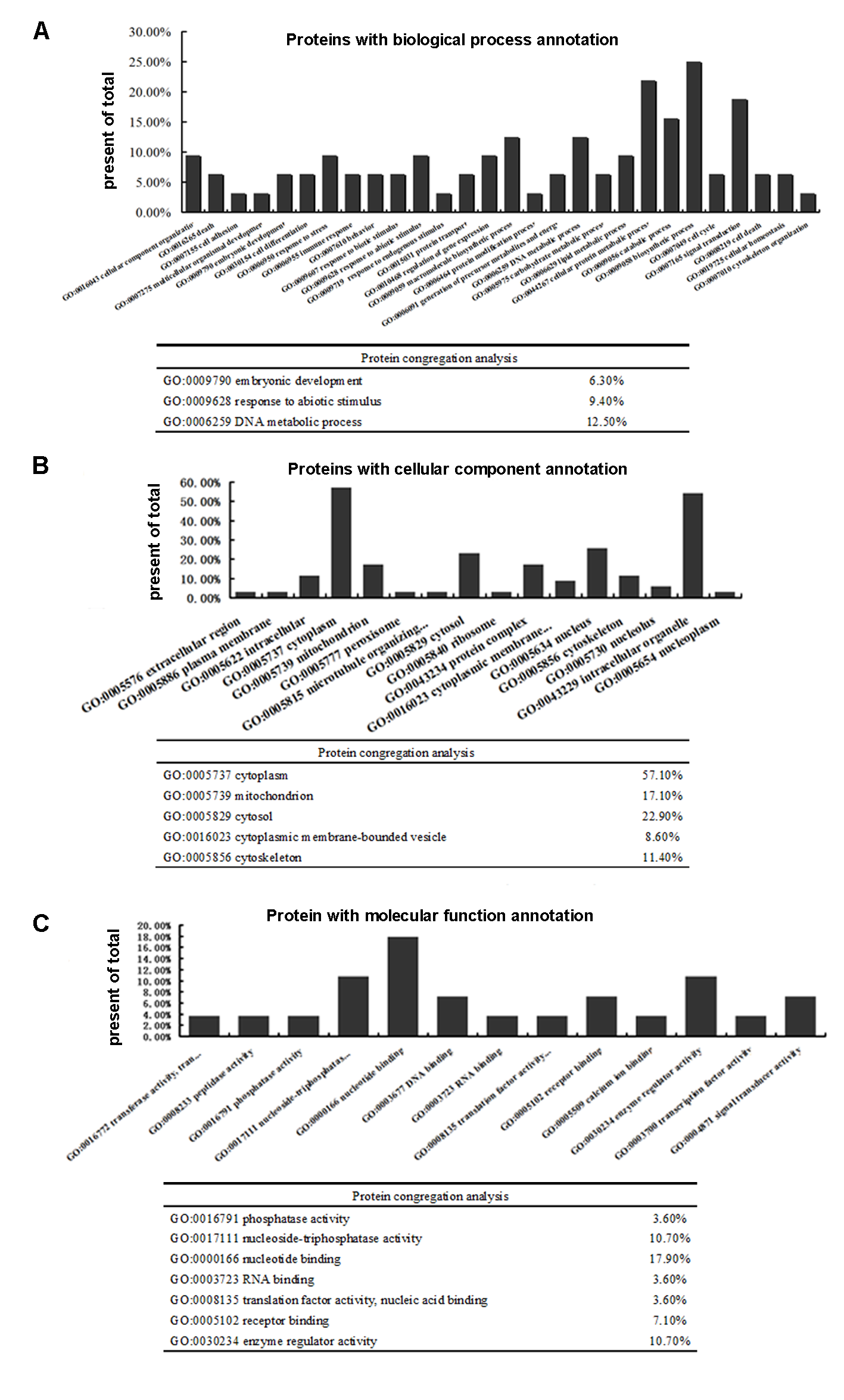

Supplement: S2 Fig — (A) Biological process annotation. (B) Cellular component annotation. (C) Molecular function annotation. (TIF) [file pone.0127568.s002.tif]

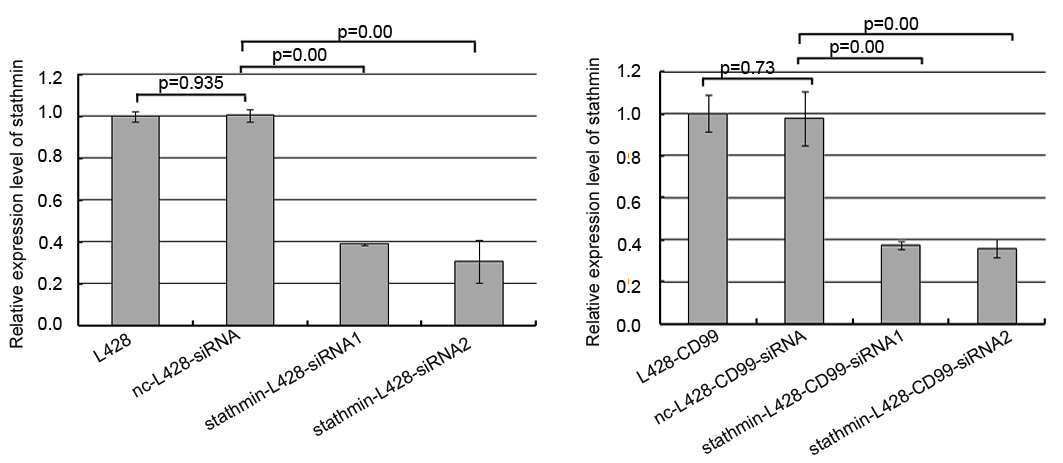

Supplement: S3 Fig — Left panel: relative expression level of STATHMIN in L428 cells transfected with STATHMIN-siRNA for 72h by qRT-PCR. Right panel: relative expression levels of STATHMIN in L428-CD99 cells transfected with STATHMIN-siRNA for 72h by qRT-PCR. (TIF) [file pone.0127568.s003.tif]
