# Supplementary material for: SEPTIN2 and STATHMIN Regulate CD99-Mediated Cellular Differentiation in Hodgkin's Lymphoma
Source: PLoS One. 2015 May 22;10(5):e0127568. doi: 10.1371/journal.pone.0127568 (PMC4441373; doi:10.1371/journal.pone.0127568)

# Supplementary material

## Materials and methods

### Cell culture and transfection

The Burkitt’s lymphoma cell line BJAB was bought from the American Type Culture Collection (ATCC, Manassas, VA, USA) and has been widely used for research (Shaohong Chen, Zhiqiang Wang, Xinzhen Dai, et al, 2013, Re-expression of microRNA-150 induces EBV-positive Burkitt lymphoma differentiation by modulating c-Myb in vitro). The cells were cultured in RPMI-1640 medium supplemented with 10% heat-inactivated fetal bovine serum (FBS) (Logan, UT, USA) at 37˚C and 5% CO_2_.

Transfection, RNA isolation, reverse transcription, and qRT-PCR analysis, and Western blotting were carried out as described in the main text. SiRNA sequence of *CD99*:

Forward: GCCAGCUGUUCAGCGUACUdTdT，

Reverse: AGUACGCUGAACAGCUGGCdTdT; *CD99* PCR primers

Forward: 5'-GCCCAGCAACAAGCAAA-GCACAT-3',

Reverse: 5'-CCCAACCACCCTAGTTCCTCCG-3'.

## Results

To further clarify the relationship between *CD99* and *STATHMIN*, we downregulated *CD99* in the L428-*CD99* cells with *CD99* siRNA and tested the expression of *CD99* and *STATHMIN* by qRT-PCR and Western blot. The results showed that silencing of *CD99* reduced the protein (Fig. 1A) and mRNA (Fig. 1B) levels of *STATHMIN* in the L428-*CD99* cells. Similarly, downregulation of *CD99* resulted in decrease of the protein (Fig. 2A) and mRNA (Fig. 2B) of *STATHMIN* in BJAB cells. These results indicated that a synergy between *CD99* and *Stathmin* expression.

**Fig. 1** Expression levels of *cd99* and *stathmin* in L428-*CD99* cells and L428-*CD99* cells transfected with *cd99* siRNA by Western blot (A), qRT-PCR (B). ※ p = 0.857, ※※p = 0.003, ★p = 0.75, ★★p = 0.000.

**Fig. 2** Expression levels of *cd99* and *stathmin* in BJAB cells and BJAB cells transfected with *cd99* siRNA by Western blot (A), qRT-PCR (B). ※ p = 0.781,※※p = 0.001, ★p = 0.604, ★★p = 0.000.


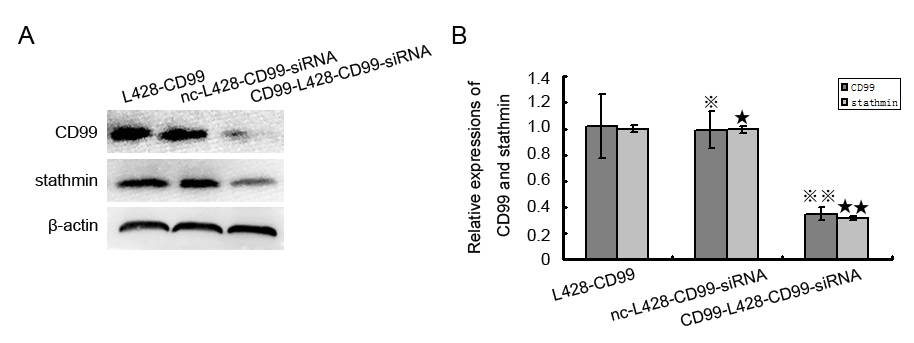

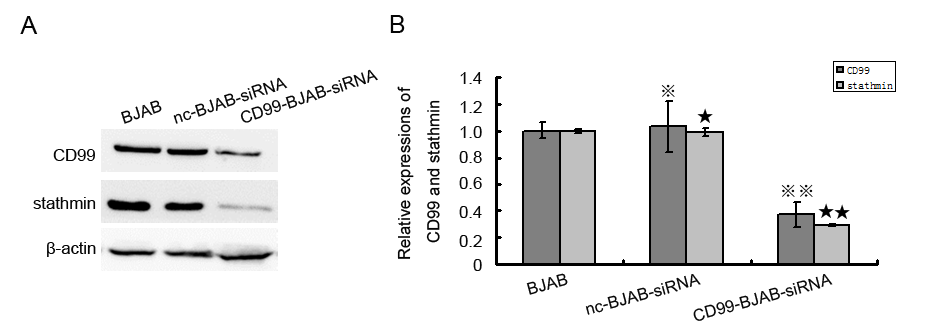

Supplement: S1 Text — (DOC) [file pone.0127568.s014.doc]
